# Seed traits inheritance in *Fagopyrum esculentum* Moench. based on image analysis method

**DOI:** 10.3389/fpls.2024.1445348

**Published:** 2024-10-09

**Authors:** Min Ah Oh, Ji Eun Park, Jae Young Kim, Ho-Min Kang, San Su Min Oh, Sheikh Mansoor, Yong Suk Chung

**Affiliations:** ^1^ Jeju Agricultural Research and Extension Services, Jeju, Republic of Korea; ^2^ National Agrobiodiversity Center, Rural Development Administration (RDA), Jeonju, Republic of Korea; ^3^ Gene Engineering Division, National Institute of Agricultural Science, Jeonju, Republic of Korea; ^4^ Interdisciplinary Program in Smart Agriculture, Kangwon National University, Chuncheon, Republic of Korea; ^5^ Department of Horticulture, Jeju National University, Jeju, Republic of Korea; ^6^ Department of Plant Resources and Environment, Jeju National University, Jeju, Republic of Korea

**Keywords:** RGB imaging, plant breeding, phenotyping, image analysis, common buckwheat

## Abstract

Common buckwheat (*Fagopyrum esculentum* Moench.) is one of the most important orphan crops worldwide. Various research efforts have been done to improve cultivation methods to enhance important agronomic traits such as productivity and biotic/abiotic resistance. One important aspect is the seed trait, which has not been extensively studied due to the time-consuming and tedious nature of its examination. Despite this, understanding seed traits is crucial for meeting consumer needs and optimizing crop yields. Therefore, the aim of the study is to investigate the inheritance of common buckwheat seed traits—such as shape, size, and coat color—using an image-based approach. This method allows for the analysis of a large number of seeds with a level of accuracy and precision that was previously unattainable. The results indicate that seed coat color is inherited maternally. Notably, the parameters in size had substantial increases acting like overdominance. The number of seeds that were harvested from F_1_s of each cross differed a lot depending on the cross combinations and pin/thrum type. In addition, seed size had large reduction in F_1_s from the different seed-sized parents, especially in thrum type. These may show that there could be cross barriers. The results revealed trends of maternal inheritance for seed shape and coat color in buckwheat, an area that has not been extensively studied. These findings could support buckwheat breeding efforts, helping to address market needs and food demands in the face of significant climate change.

## 1 Introduction

Common buckwheat (*Fagopyrum esculentum* Moench.) has a short growing season and can thrive in poor soils and mountainous regions worldwide (Chrungoo and Chettry, 2021). However, its yield is lower compared to other crops such as rice and maize (Kim et al., 2017). Factors contributing to this low yield include lodging, shattering, and infertility (Farooq et al., 2016). Consequently, breeding efforts primarily focus on developing varieties with lodging resistance and shattering tolerance. Research also targets infertility alleviation, with studies focusing on quantitative traits of the plant such as plant length, stem thickness, and panicle size (Suzuki et al., 2023), along with investigations into infertility mitigation (Inoue et al., 2002).

Tartary buckwheat seeds are a valuable resource, widely utilized in the production of various food products (Ma et al., 2013; Merendino et al., 2014; Qin et al., 2013). As these seeds mature, they undergo significant changes in shape, color, and dry weight. Anthocyanins, the pigments responsible for the coloration of fruits, leaves, and flowers, are integral to this process (Harborne, 1963). Studies have shown that different anthocyanins accumulate at various stages of buckwheat flower development (Suzuki et al., 2007). Furthermore, the anthocyanin content in Tartary buckwheat sprouts is influenced by exposure to specific light and dark cycles (Peng et al., 2015).

Buckwheat breeding efforts primarily focus on enhancing seed yield and improving the quality of cultivated common buckwheat. Currently, the average yields for common and Tartary buckwheat are approximately 1,050 kg/ha and 1,800 kg/ha, respectively, with maximum yields reaching up to 2,500 kg/ha and 3,000 kg/ha (Luthar et al., 2021). In contrast, all natural perennial buckwheat species remain wild and exhibit several undesirable traits, including seed shattering, high sensitivity to photoperiods and temperature, indeterminate flower and fruit development, a non-compact growth habit, low fertility and yield, and strong seed dormancy (Chen et al., 2018). Seed characteristics of common buckwheat, including shape, size, and color, play a crucial role in post-processing quality, consumer preferences, and overall yield. Common buckwheat seeds exhibit various shapes, typically characterized by a trullate cross section (Ahmed et al., 2014). This morphological trait can significantly impact post-processing quality. The degree of damage during refining processes, such as de-hulling and milling, varies based on seed length and width, which in turn affects yield and quality (Trotsenko et al., 2021). Additionally, seed size influences not only threshing efficiency (Tetsuka and Uchino, 2005) but also yield determination in buckwheat (Li et al., 2019). Larger seeds generally contribute to heavier yields, leading to higher overall production.

Numerous studies have investigated germplasm diversity using molecular techniques, especially next-generation sequencing technologies (Dar et al., 2018; Yasui, 2020). These investigations have revealed significant genetic variations and traced evolutionary lineages, offering valuable insights for optimizing breeding strategies (Dar et al., 2018; Yabe and Iwata, 2020). While understanding genotype-by-environment interactions is vital for breeding programs targeting extensive regions, the use of molecular data significantly enhances this process. Nonetheless, local phenotypic characteristics remain essential for breeding efforts that are specific to particular environments (Karunathilake et al., 2023; Sheikh et al., 2024; Han et al., 2024; Mansoor and Chung, 2024; Mansoor et al., 2024).

Research on buckwheat seed size has delved into genes associated with seed size and the timing of seed size determination (Fang et al., 2019), as well as the genetic factors influencing seed weight (Li et al., 2012). Buckwheat seed coat color varies widely, ranging from white, gray, and light brown to dark brown and black (Rauf et al., 2020). Preferences for seed coat color differ among processing companies and consumers (Inoue et al., 2002; Yoshida et al., 1995). However, studies on the inheritance of seed characteristics in common buckwheat for breeding purposes remain limited. Thus, the current study investigated the genetic inheritance on shape, size, and color in common buckwheat seeds in F_1_ of parental combination of various morphological traits using image analysis approach. Image analysis could offer an accurate and objective method for measuring and evaluating seed traits like size, shape, and color. High-throughput approach is essential for identifying desirable characteristics and speeding up breeding programs and the seed attributes such as size, shape, and color can have a direct impact on the post-processing quality of buckwheat products.

## 2 Materials and methods

### 2.1 Plant materials

**Figure 1:**
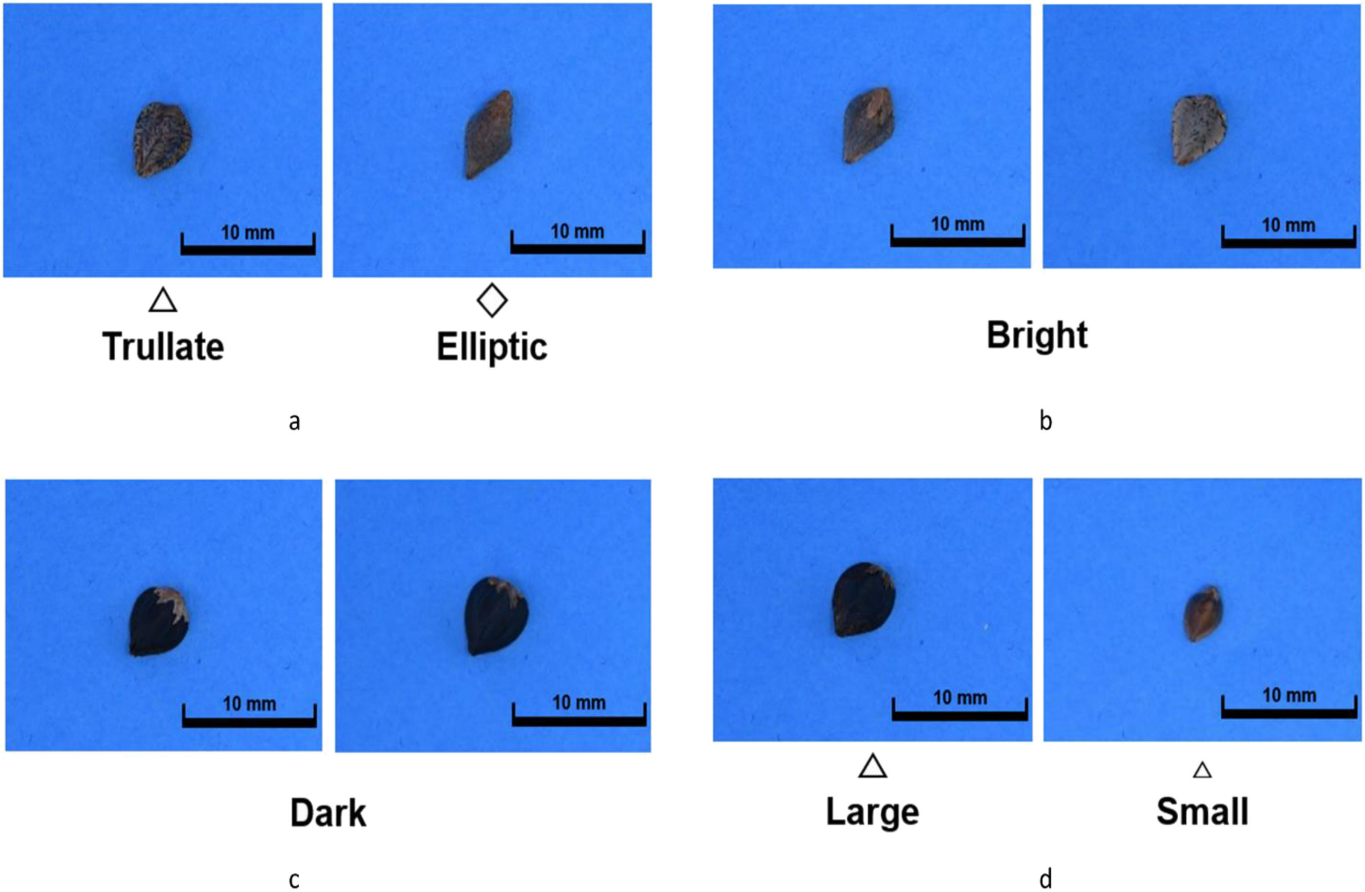
The seed images with morphological parameters. **(A)** is the shape group, which consisted of “trullate” and “elliptic” seed shape. **(B)** is the “Bright” seed coat color. **(C)** is the “Dark” seed coat color. **(D)** is the seed size group, consisted of “Large” and “Small”.

We obtained around 100 buckwheat germplasms from the National Agrobiodiversity Center at the National Institute of Agricultural Sciences. Initially, these were classified into two primary groups based on seed color: dark and bright colored. Subsequently, they were further categorized into trullater and elliptic groups based on seed shape. One representative germplasm was selected from each group, and within each selected germplasm, 10–18 seeds with the most distinct characteristics were chosen. Each individual was given a unique identifier (**Figure 1**). Seed shape and color classification resulted in four groups in total: dark-colored seed coat with elliptic seeds (DD), dark-colored seed coat with trullater seeds (DT), bright-colored seed coat with elliptic seeds (BD), and bright-colored seed coat with trullater seeds (BT). Additionally, to observe the phenotype of F_1_ hybrids concerning size, germplasms representing alternative/large sized (LS) and small (SS) sized seeds were selected alongside the aforementioned four groups. As a result, six germplasms that best represented the traits among the 100 germplasms were chosen for experimentation (**Table 1**).

**Table 1:** The germplasm IDs of each phenotype groups.

Phenotype groups	Germplasm ID	Abbreviations	Number of seeds
Bright-colored seed coat and trullate shape	IT288930	BT	114
Bright-colored seed coat and elliptic shape	IT310552	BD	227
Dark-colored seed coat and trullate shape	IT318103	DT	76
Large size	IT288929	LS	100
Small size	IT318104	SS	6

### 2.2 Crosses

**Figure 2:**
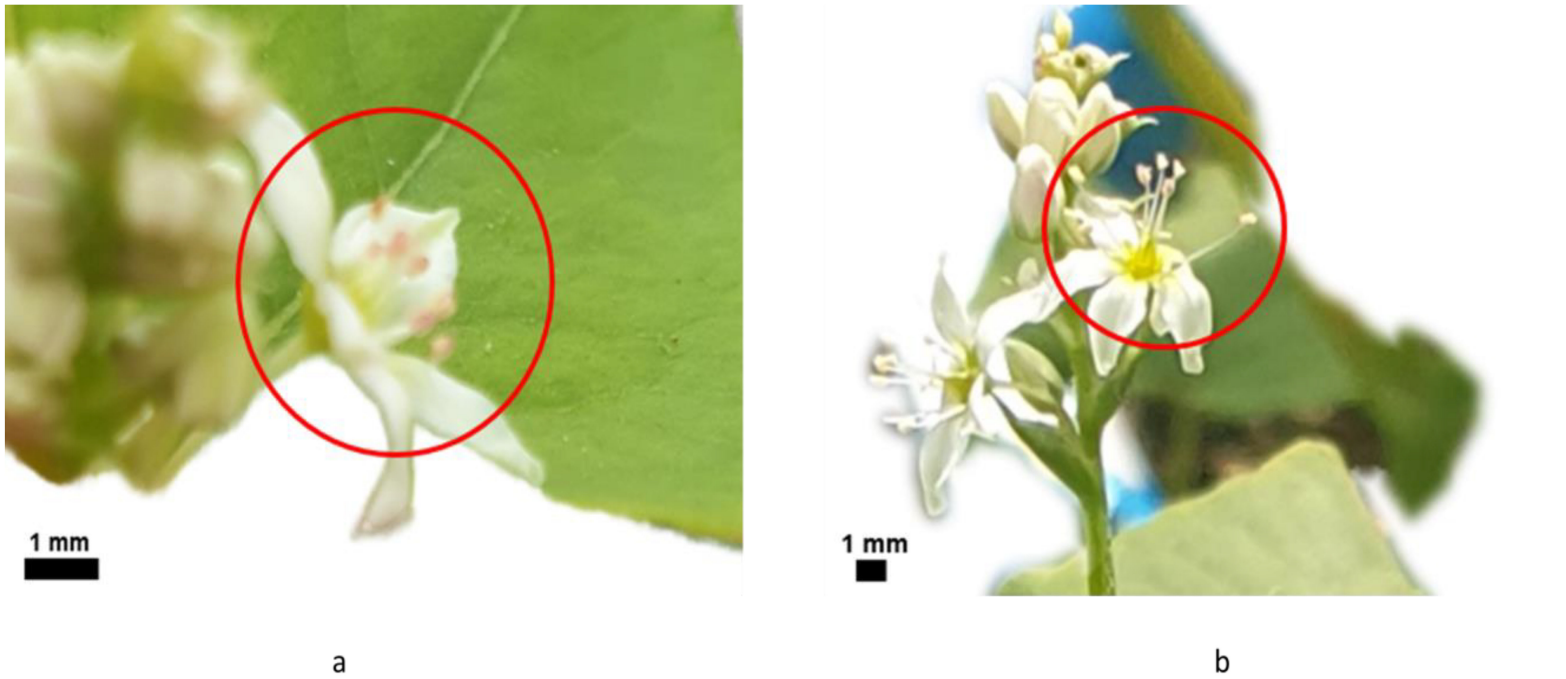
The floral type of common buckwheat by their characteristics of floral parts. **(A)** is the image of pin-type flower, which has long pistil and short stamens. **(B)** is the image of thrum-type flower, which has short pistil and long stamens.

Seeding was conducted on 31 December 2022 at the Glass Greenhouse of Jeju National University (Jeju, Republic of Korea). Soil (Bio Soil, Heungnong, Republic of Korea) was filled to 80% of 15 cm diameter pots, and each pot was sown with one seed, with the unique identifier marked on the pot. To manage the growth of the seeds, moisture control was implemented to prevent soil from drying out. Upon flowering, the determination of long-styled and short-styled flowers was made based on the length of the styles (**Figure 2**), and the flowers that bloomed prematurely were removed before isolation according to the experimental design because only different types of styles can be crossed (Cawoy et al., 2009). Healthy individuals were appropriately arranged according to the experimental design. However, seeds with dark colored seed coat and elliptic shape (DD) did not germinate and were not utilized for the experiment. The crosses were made as follows (**Table 2**):

**Table 2:** Summary of F_1_s in each group combination.

Phenotypes	Crossed groups and types	Germplasm IDs of F_1_ seeds	Number of seeds (pin, thrum)
Seed shape	BT (pin) × BD (thrum) BD (pin) × BT (thrum)	IT288930-(13) × IT310552-(8) IT310552-(2) × IT288930-(2)	116 (20, 96) 151 (131, 20)
Seed coat color	DT (pin) × BT (thrum) BT (pin) × DT (thrum)	IT318103-(7) × IT288930-(11) IT288930-(15) × IT318103-(6)	126 (62, 64) 24 (10, 14)
Seed size	LS (pin) × SS (thrum) SS (pin) × LS (thrum)	IT288929-(9) × IT318104-(3) IT318104-(7) × IT288929-(4)	8 (7, 1) 98 (5, 93)

- After BT and BD were crossed, the seed shape of F_1_ that was harvested from each mother plant was compared.
- After DT and BT were crossed, the seed coat color of F_1_ that was harvested from each mother plant was compared.
- After LS and SS were crossed, the size of F_1_ that was harvested from each mother plant was compared.

Additionally, to explore whether the flower form affects the seed form, both long-styled and short-styled flowers were used in the parent plants for each combination. In total, eight combinations were studied, with each isolated and subjected to fly irradiation for modification. The seeds harvested from the mature plants were then stored.

### 2.3 Image acquisition and analysis

**Figure 3:**
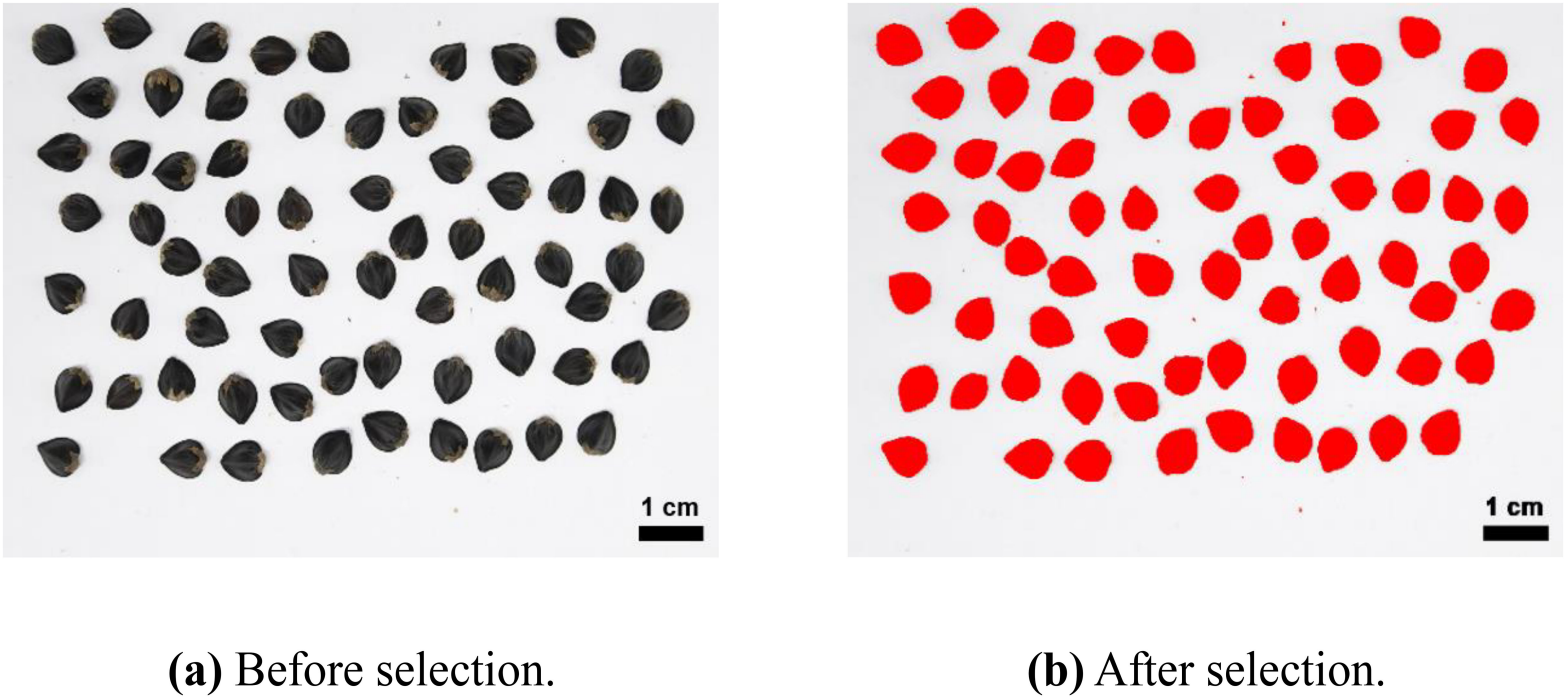
Background separation and area selection using the color-thresholding tool in ImageJ. **(A)** is the raw buckwheat image before background separation. **(B)** is the buckwheat image after background separation. Red area is ROIs (region of interests) selected through color thresholding.

The images of seeds before and after harvesting were acquired using an RGB camera (Nikon, Japan, EOS D200II, Lens EF-S 18-55mm) and captured in the Image Studio installed in Jeju National University’s Crop Breeding and Cultivation Laboratory (**Supplementary Figure S1**). The captured images were analyzed using open-source software ImageJ (ver. 1.54f) (Schneider et al., 2012) as shown in **Figure 3**. Various variables were calculated to analyze the shape of the seeds, including aspect ratio (the ratio of the longest length to the shortest width).

Seed size was determined using parameters such as area, length, and width, measured in pixels (**Table 3**). The color of the seed coat was analyzed by extracting pixel data from the red, green, and blue bands of the images; converting them to grayscale; and using the representative color of each seed (Padmavathi and Thangadurai, 2016). Grayscale conversion follows the formula:

**Table 3:** The parameters and terms used in present image analysis.

Parameters	Definitions
Area	The total number of pixels in the selected region.
Length	The number of pixels in the vertical width of the selected region.
Width	The number of pixels in the horizontal width of the selected region.
Aspect ratio	The ratio of the longest length to the shortest width.


(1) Grayscale (Y)=0.2989R+0.570G+0.1140B

Y represents the grayscale value, R is the value of the red band in the representative color, G is the value of the green band, and B is the value of the blue band. Correlation analysis of R, G, and B revealed a high positive correlation among them ranging from 0.87 to 0.95. For bright-colored seed coat, all three bands had higher values compared to dark-colored seed coat, which justifies converting grayscale.

### 2.4 Statistical analysis

The data extracted from the images were statistically analyzed using RStudio (ver. 4.3.0). After removing outliers, the data were normalized. To assess normality, Shapiro test was conducted, confirming normal distribution of the data. However, Bartlett test revealed heteroscedasticity, indicating unequal variances among the groups. Therefore, Welch’s t-test was used to analyze the differences between the measurements of F_1_ for each combination. In cases where the sample size for comparing seed size was insufficient, Wilcoxon rank-sum test was used for analysis. Moreover, the Kruskall–Wallis test was carried out to investigate the differences in each phenotype groups and parameters, and Dunn’s test was implemented for *post-hoc* test.

## 3 Results

### 3.1 Seed shape

**Figure 4:**
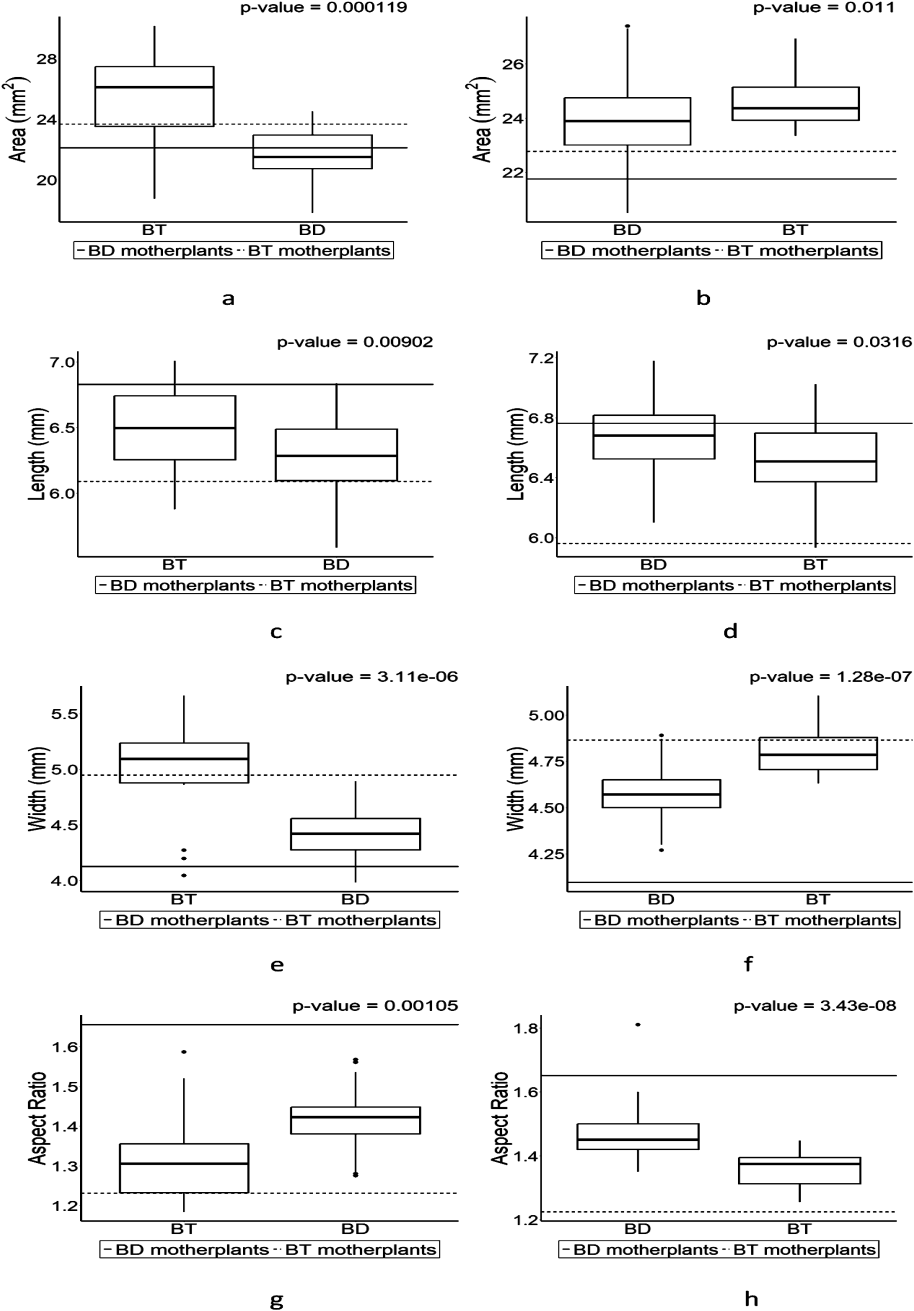
The boxplot for seed shapes in F_1_ progenies which were derived from the crosses of trullate-shape seed-bearing individual (BT) and elliptic-shape seed-bearing individual (BD) individuals. Values above the plot is the results of Wilcoxon rank-sum test. The boxplot on the left is the pin-type flower, and the boxplot on the right side is the thrum-type flower. Horizontal lines represent parental parameter value of each boxplot. **(A)** is the area of BT (pin) and BD (thrum). **(B)** is the area of BD (pin) and BT (thrum). **(C)** is the length of BT (pin) and BD (thrum). **(D)** is the length of BD (pin) and BT (thrum). **(E)** is the width of BT (pin) and BD (thrum). **(F)** is the width of BD (pin) and BT (thrum). **(G)** is the aspect ratio of BT (pin) and BD (thrum). **(H)** is the aspect ratio of BD (pin) and BT (thrum).

Four parameters including length, width, area, and aspect ratio (AR) were used for comparison for seed shape of F_1_ seeds from each cross (**Figure 4**). The patterns of the values in each parameter from F_1_ seeds follow its parental character except in length of F_1_ from BT and BD when BT is a pin type (**Figure 4C**). Slight increases were observed in area of BT with exceeded values to parental values in length while the values in width remained similar no matter what types BT is (**Figures 4A, C, E**). Unlike that of BT, BD showed different patterns in length and width depending on its types (**Figures 4B, D, F**). BT had exceeded value and BD had lower value in height compared to its parental values when BT is pin type (**Figures 4C, D**), while BD had exceeded values in width in both pin and thrum types unlike BT (**Figures 4E, F**), although length and width in F_1_s were within their parental values. Nonetheless, trullater-shaped seeds-bearing individual tend to have trullater-shaped offspring and elliptic-shaped seed-bearing individual tends to produce elliptic-shaped offspring although AR values in F_1_s compared to their parental ARs are close each other no matter what their types are (**Figures 4G, H**).

### 3.2 Seed size

**Figure 5:**
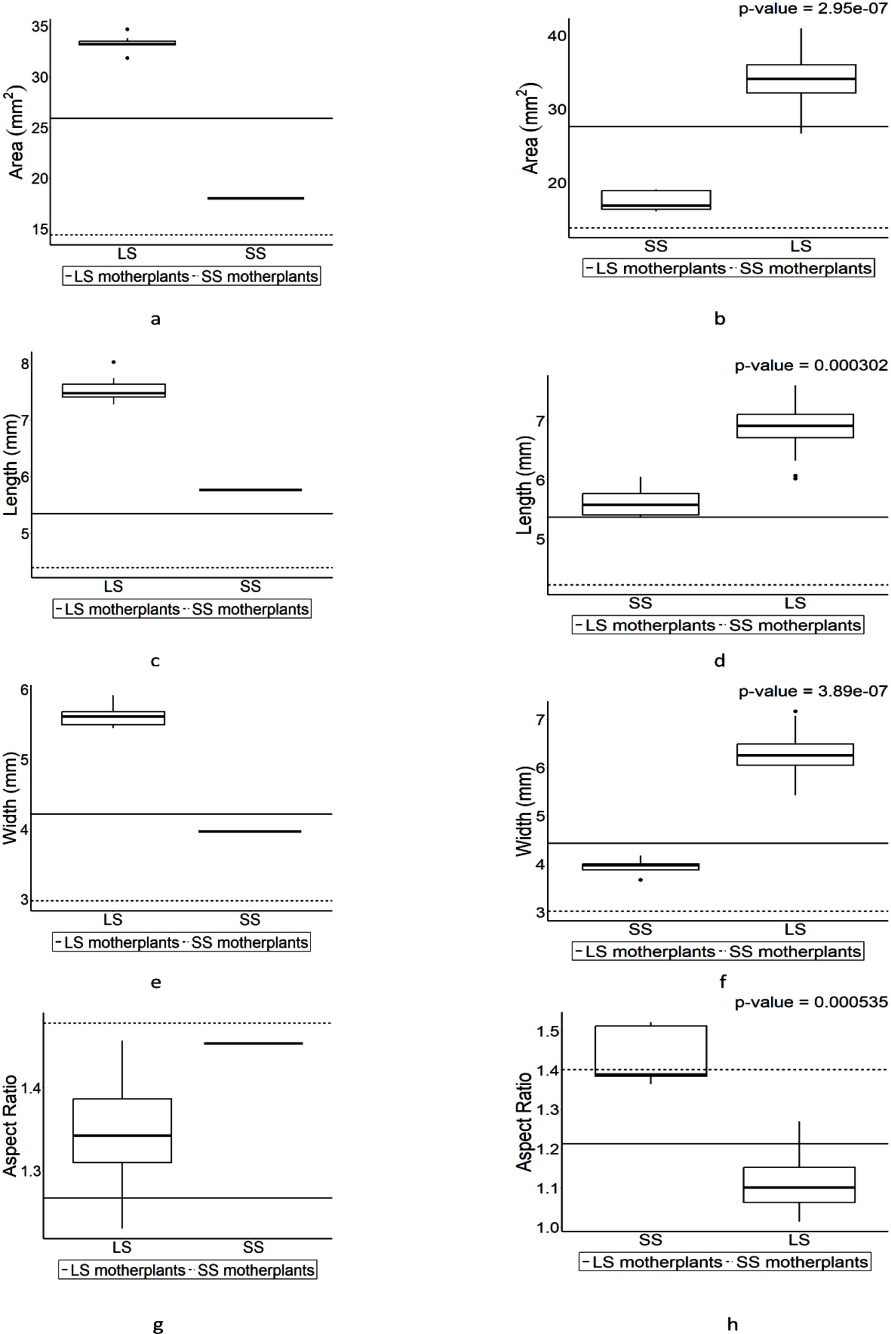
The boxplot for seed size in F_1_ progenies which were derived from the crosses of large seed-bearing (LS) and small seed-bearing (SS) individuals. Values above the plot are the results of Wilcoxon rank-sum test. The boxplot on the left side is the pin-type flower, and the boxplot on the right side is the thrum-type flower. Horizontal lines represent parental parameter value of each boxplot. **(A)** is the area of LS (pin) and SS (thrum). **(B)** is the area of SS (pin) and LS (thrum). **(C)** is the length of LS (pin) and SS (thrum). **(D)** is the length of SS (pin) and LS (thrum). **(E)** is the width of LS (pin) and SS (thrum). **(F)** is the width of SS (pin) and LS (thrum). **(G)** is the aspect ratio of LS (pin) and SS (thrum). **(H)** is the aspect ratio of SS (pin) and LS (thrum).

The values of area, height, and width in F_1_s exceeded their parental values in both pin and thrum types (**Figures 5A–F**). Notably, the values of F_1_s in length from the crosses both between LS and SS and between SS and LS exceeded significantly (**Figures 5C, D**) while the values of SS in area and width remained within the parental values (**Figures 5A, B, E, F**). Since the rates of increases in each case differ, the AR pattern changed accordingly in F_1_s (**Figures 5G, F**).

### 3.3 Seed coat color

**Figure 6:**
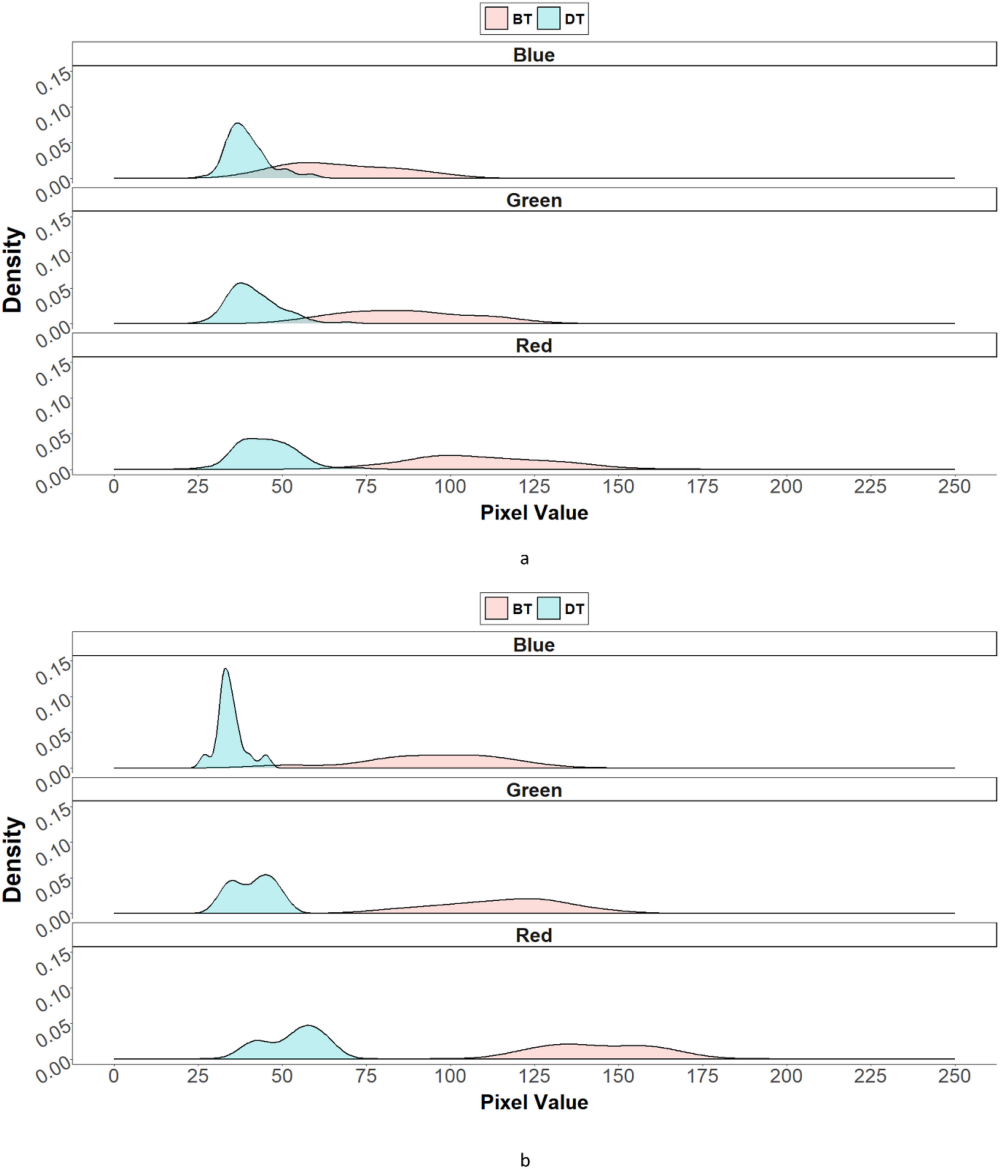
The distribution density plot of red, green, and blue values based on seed color in F_1_ progenies, which were derived from the crosses of dark-colored seed-bearing individual (DT) and bright-colored seed-bearing individual (BT) individual. **(A)** is the RGB distribution density of DT (pin) and BT (thrum). **(B)** is the RGB distribution density of BT (pin) and DT (thrum).

The red (R), green (G), and blue (B) bands and their distributions from RGB images are shown in **Figure 6**. The distribution of R, G, and B in dark seeds while that in bright seeds tend to cluster one another.

**Figure 7:**
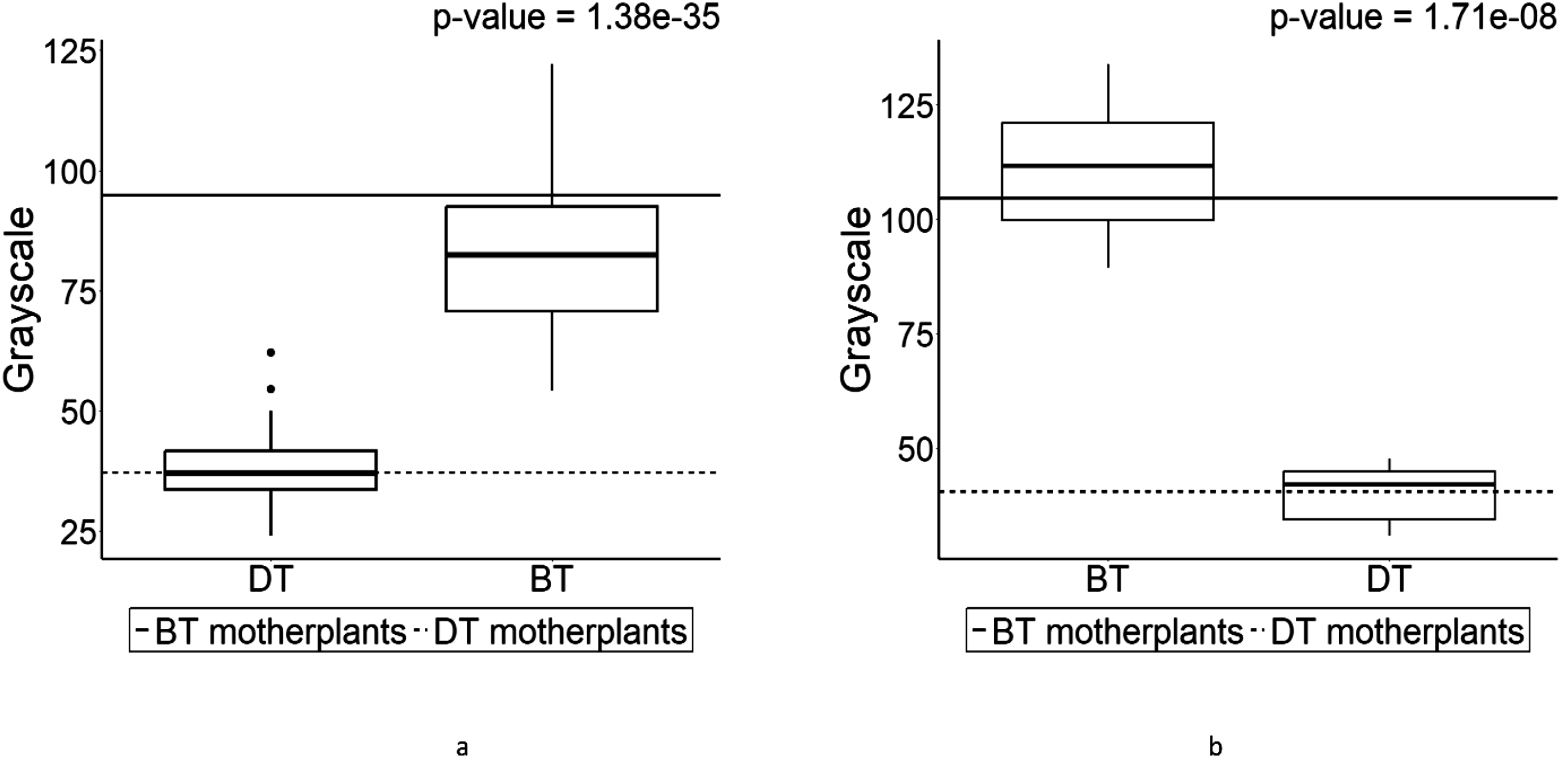
The boxplot of grayscale in F_1_ progenies, which were derived from the crosses of dark colored seed-bearing individual (DT) and bright colored seed-bearing individual (BT) individuals. Values above the plot are the results of Wilcoxon rank-sum test. The boxplot on the left side is the pin-type flower, and the boxplot on the right side is the thrum-type flower. Horizontal lines in represent parental parameter value of each boxplot. **(A)** is the grayscale of DT (pin) and BT (thrum). **(B)** is the grayscale of BT (pin) and DT (thrum).

Based on this difference, the grayscale values were calculated to make comparisons in seed coat color (**Figure 7**). The seed coat colors in F_1_s from each cross (**Figures 7A, B**) were almost identical to their parental values implying seed coat color inheritance follows maternally although there are little variances. Kruskall–Wallis test for each F1 phenotype group is presented in **Table 4**. While as analysis of variance table, showing degree of freedom and *p*-values as well as the results of post-hoc test (Dunn test) are in **Supplementary Tables S1**-**S3**.

**Table 4:** Kruskall–Wallis test for each F_1_ phenotype group.

Groups	Parameters	*df*	Chi-squared	*p*-value
Seed shape	Area	3	105.03	< 0.001^*^
	Height	3	89.341	< 0.001^*^
	Width	3	96.621	< 0.001^*^
	Aspect ratio	3	71.807	< 0.001^*^
Seed size	Area	3	17.342	< 0.001^*^
	Height	3	31.393	< 0.001^*^
	Width	3	30.163	< 0.001^*^
	Aspect ratio	3	33.75	< 0.001^*^
Seed coat color	Grayscale	3	115.76	< 0.001^*^

^*^Indicates statistically significant differences (p < 0.01).

## 4 Discussion

The inheritance of seed traits (color) is crop specific; some are maternal and the others are paternal. Maize (*Zea mays* L.) (Ron-Parra et al., 2016; Wright and Neuffer, 1989), sesame (*Sesamum indicum*), rapeseed (*Brassica napus*) (Rahman et al., 2010), and cowpea (*Vigna unguiculata*) (Ajayi et al., 2020) follow maternal side while that of wheat (*Triticum aestivum*) depends on environmental factors, indicating that seed coat color in all plants does not necessarily follow maternal inheritance (Calderon Flores et al., 2021).

The seed coat is the outer protective layer of a seed, formed from the integuments of the ovule after fertilization. It serves to protect the seed from physical damage, dehydration, and sometimes from predators and pathogens (Copeland and McDonald, 2012). The seed coat plays a crucial role in the seed’s viability and germination process. Seeds are formed by process of double fertilization (Boesewinkel and Bouman, 1984). Moreover, Leon-Kloosterziel et al., 1994 reported that seed shape in *Arabidopsis thaliana* is maternally determined by the seed coat. Furthermore, Naseem et al. (2007) found that traits such as seed shape, size, and seed coat color have high heritability compared to other quantitative traits. Fang et al. (2012) discovered that seed size in *Arabidopsis thaliana* is maternally inherited through several genes. According to Jiang and Lin (2013), the plant hormone brassinosteroid (BR) regulates seed length in endosperm tissues, including the seed coat, and sufficiently influences seed size in the outer integument. These findings might explain that the variance of each boxplot in **Figures 4**, **5**, **7** was small as a result of qualitative inheritance, although it varied in the current study. It should be investigated further to find out if it is quantitative or quantitative inheritance in F_2_ generation based on heritability.

Similarly, Lemontey et al., 2000 observed strong maternal inheritance of seed size in pea (*Pisum sativum* L.) when intercrossing different-sized parental accessions, with F_1_ generation seed sizes following maternal inheritance. Singh et al. (2017) found that when different varieties of bean plants (*Phaseolus vulgaris* L.) were intercrossed, the resulting F1 generation seeds closely resembled the parent seeds in both shape and size. This suggests that these traits are strongly inherited from the parent plants. Similarly, Zhang et al. (2016) observed that when maize plants with significantly different parental traits were intercrossed, the seed weight and size showed strong maternal inheritance. This means that the traits of seed weight and size were predominantly influenced by the mother plant’s genetics, rather than being equally influenced by both parents. The results in the current study shows that seed coat color is inherited maternally in common buckwheat as previous studies. However, the parameters in shape and size seems to have interactions with paternal side to have variances. Among these variances, the parameters in size had substantial increases acting like overdominance, especially in length unlike other reports. This may be determined by different interactions among genes located outside the maternal parent’s nucleus (Li and Li, 2015).

Given the rarity of studies on the inheritance of seed traits in common buckwheat, this research provides valuable insights into the genetic inheritance of seed shape, size, and color. These findings can be leveraged for breeding purposes. Interestingly, the number of seeds harvested from each cross varied significantly, suggesting potential cross barriers within certain combinations. This variability may reflect barriers between contrasting phenotypes or different genetic backgrounds from distinct germplasms.

Future research should explore cross barriers more comprehensively by examining a broader range of cross combinations and increasing replication efforts. The seed size exhibits a unique pattern of overdominance not observed in other crops, which warrants further investigation across multiple generations beyond the F_2_ stage. Additionally, integrating advanced imaging technologies with machine learning and artificial intelligence could significantly enhance trait analysis. Investigating how seed traits interact with environmental factors and stress conditions will be crucial for developing high-yielding and climate-resilient varieties. Combining phenotypic data with genomic information will improve breeding programs by refining selection decisions and accelerating the development of new buckwheat varieties with desirable traits.

## 5 Conclusions

Breeding efforts in common buckwheat primarily have focused on improving growth characteristics and acquiring resistance to biotic/abiotic stresses. Thus, the inheritance of common buckwheat seed traits was examined for the first time based on image analysis, which allows us to examine lots number of seeds with high accuracy and precision unlike before. This study revealed that the inheritance modes of shapes and colors in seeds seem to be similar with the previous studies while that of size exhibits overdominance unlike other crops. This may need to be investigated further through generations beyond the F_2_ stages. Nevertheless, given that the inheritances of seed characters of common buckwheat are rare as mentioned above, the current study showed the genetic inheritance on shape, size, and color in common buckwheat seeds using image analysis. These would be useful in common buckwheat for breeding purposes.

## Data Availability

The datasets presented in this study can be found in online repositories. The names of the repository/repositories and accession number(s) can be found in the article/**Supplementary Material**.
