# Supplementary material for: Seed traits inheritance in Fagopyrum esculentum Moench. based on image analysis method
Source: Front Plant Sci. 2024 Oct 9;15:1445348. doi: 10.3389/fpls.2024.1445348 (PMC11496140; doi:10.3389/fpls.2024.1445348)
Supplement: Supplementary file 1 [file DataSheet1.docx]

| 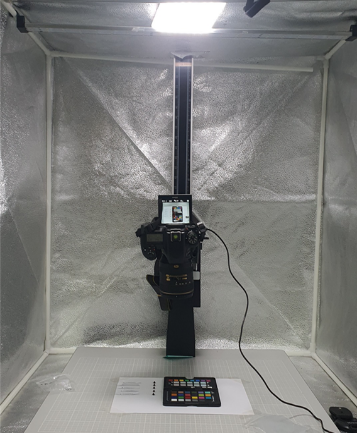 | 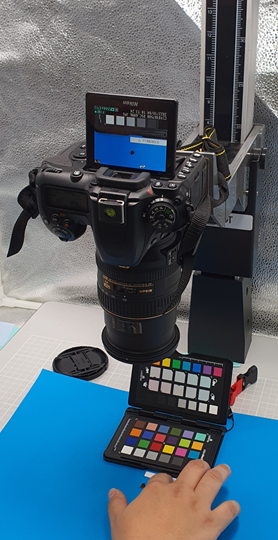 |
| --- | --- |
| **(a)** | **(b)** |

**Supplementary Fig. 1.** The Image Studio of the Crop Breeding and Cultivation Laboratory at Jeju National University. (a) is an overall image of an imaging studio. It consists of a digital camera for image acquisition, an adjustable height camera tripod, lights and reflective tents to minimize shadows, and a color checker for color calibration. (b) is an image showing a blue background that facilitates easy background separation using the Lab color space during video shooting, along with a photo of the color checker.

**Supplementary Table 1.** Dunn test for F_1_ Shape phenotype group as post-hoc test for Kruskall-Wallis test.

| Parameters | Comparison | | Z | unadjusted p-values | adjusted p-values |
| --- | --- | --- | --- | --- | --- |
| Area | IT288930-(13) - | IT288930-(2) | -0.0767 | 0.9388 | 0.9388 |
|  | IT288930-(13) - | IT310552-(2) | 1.7125 | 0.0868 | 0.1736 |
|  | IT288930-(2) - | IT310552-(2) | 1.8137 | 0.0697 | 0.2092 |
|  | IT288930-(13) - | IT310552-(8) | 6.4463 | < 0.0001 | < 0.0001^*^ |
|  | IT288930-(2) - | IT310552-(8) | 6.5451 | < 0.0001 | < 0.0001^*^ |
|  | IT310552-(2) - | IT310552-(8) | 8.7335 | < 0.0001 | < 0.0001^*^ |
| Height | IT288930-(13) - | IT288930-(2) | -0.1454 | 0.8844 | 0.8844 |
|  | IT288930-(13) - | IT310552-(2) | -2.2084 | 0.0272 | 0.0816 |
|  | IT288930-(2) - | IT310552-(2) | -2.0169 | 0.0437 | 0.0874 |
|  | IT288930-(13) - | IT310552-(8) | 3.0080 | 0.0026 | 0.0105^*^ |
|  | IT288930-(2) - | IT310552-(8) | 3.1950 | 0.0014 | 0.0070^*^ |
|  | IT310552-(2) - | IT310552-(8) | 9.4493 | < 0.0001 | < 0.0001^*^ |
| Width | IT288930-(13) - | IT288930-(2) | -0.2416 | 0.8091 | 0.8091 |
|  | IT288930-(13) - | IT310552-(2) | 4.1069 | < 0.0001 | < 0.0001^*^ |
|  | IT288930-(2) - | IT310552-(2) | 4.4252 | < 0.0001 | < 0.0001^*^ |
|  | IT288930-(13) - | IT310552-(8) | 7.2010 | < 0.0001 | < 0.0001^*^ |
|  | IT288930-(2) - | IT310552-(8) | 7.5119 | < 0.0001 | < 0.0001^*^ |
|  | IT310552-(2) - | IT310552-(8) | 5.8358 | < 0.0001 | < 0.0001^*^ |
| Aspect ratio | IT288930-(13) - | IT288930-(2) | -0.1116 | 0.9111 | 0.9111 |
|  | IT288930-(13) - | IT310552-(2) | -6.1756 | < 0.0001 | < 0.0001^*^ |
|  | IT288930-(2) - | IT310552-(2) | -6.0285 | < 0.0001 | < 0.0001^*^ |
|  | IT288930-(13) - | IT310552-(8) | -3.3629 | 0.0007 | 0.0023^*^ |
|  | IT288930-(2) - | IT310552-(8) | -3.2193 | 0.0012 | 0.0026^*^ |
|  | IT310552-(2) - | IT310552-(8) | 4.8825 | < 0.0001 | < 0.0001^*^ |

^*^ Indicates statistically significant differences (p < 0.01).

**Supplementary Table 2.** Dunn test for F_1_ Size phenotype group as post-hoc test for Kruskall-Wallis test.

| Parameters | Comparison | | Z | unadjusted p-values | adjusted p-values |
| --- | --- | --- | --- | --- | --- |
| Area | IT288929-(4) - | IT288929-(9) | 0.7203 | 0.4713 | 0.9427 |
|  | IT288929-(4) - | IT318104-(3) | 1.7183 | 0.0857 | 0.3430 |
|  | IT288929-(9) - | IT318104-(3) | 1.3518 | 0.1764 | 0.5293 |
|  | IT288929-(4) - | IT318104-(7) | 3.8054 | 0.0001 | 0.0008^*^ |
|  | IT288929-(9) - | IT318104-(7) | 2.5014 | 0.0124 | 0.0618 |
|  | IT318104-(3) - | IT318104-(7) | 0.0178 | 0.9858 | 0.9858 |
| Height | IT288929-(4) - | IT288929-(9) | -3.8318 | 0.0001 | 0.0006 |
|  | IT288929-(4) - | IT318104-(3) | 1.5937 | 0.1110 | 0.2220 |
|  | IT288929-(9) - | IT318104-(3) | 2.9036 | 0.0037 | 0.0111^*^ |
|  | IT288929-(4) - | IT318104-(7) | 3.5184 | 0.0004 | 0.0017^*^ |
|  | IT288929-(9) - | IT318104-(7) | 5.3234 | < 0.0001 | < 0.0001^*^ |
|  | IT318104-(3) - | IT318104-(7) | 0.0119 | 0.9905 | 0.9905 |
| Width | IT288929-(4) - | IT288929-(9) | 3.6524 | 0.0003 | 0.0013^*^ |
|  | IT288929-(4) - | IT318104-(3) | 1.7982 | 0.0721 | 0.2886 |
|  | IT288929-(9) - | IT318104-(3) | 0.3521 | 0.7248 | 1.0000 |
|  | IT288929-(4) - | IT318104-(7) | 3.9805 | < 0.0001 | 0.0004^*^ |
|  | IT288929-(9) - | IT318104-(7) | 0.6761 | 0.4990 | 1.0000 |
|  | IT318104-(3) - | IT318104-(7) | 0.0178 | 0.9858 | 0.9858 |
| Aspect ratio | IT288929-(4) - | IT288929-(9) | -4.2072 | 0.0003 | 0.0002^*^ |
|  | IT288929-(4) - | IT318104-(3) | -1.8112 | 0.0701 | 0.2804 |
|  | IT288929-(9) - | IT318104-(3) | -0.1608 | 0.8722 | 1.0000 |
|  | IT288929-(4) - | IT318104-(7) | -3.9097 | 0.0001 | 0.0004^*^ |
|  | IT288929-(9) - | IT318104-(7) | -0.2492 | 0.8032 | 1.0000 |
|  | IT318104-(3) - | IT318104-(7) | 0.0238 | 0.9810 | 0.9810 |

^*^ Indicates statistically significant differences (p < 0.01).

**Supplementary Table 3.** Dunn test for F_1_ Color phenotype group as post-hoc test for Kruskall-Wallis test.

| Parameters | Comparison | | Z | unadjusted p-values | adjusted p-values |
| --- | --- | --- | --- | --- | --- |
| Grayscale | IT288930-(11) - | IT288930-(15) | -2.0011 | 0.0454 | 0.0908 |
|  | IT288930-(11) - | IT318103-(6) | 4.9991 | < 0.0001 | < 0.0001^*^ |
|  | IT288930-(15) - | IT318103-(6) | 5.2058 | < 0.0001 | < 0.0001^*^ |
|  | IT288930-(11) - | IT318103-(7) | 9.3525 | < 0.0001 | < 0.0001^*^ |
|  | IT288930-(15) - | IT318103-(7) | 6.8873 | < 0.0001 | < 0.0001^*^ |
|  | IT318103-(6) - | IT318103-(7) | 0.6576 | 0.5173 | 0.5726 |

^*^ Indicates statistically significant differences (p < 0.01).
